# Vaccination in the childhood and awareness of basic public health services program among internal migrants: a nationwide cross-sectional study

**DOI:** 10.1186/s12889-023-16147-z

**Published:** 2023-06-28

**Authors:** Jun Wang, Yang Bai, Jingmin Zhu, Xueyao Wang, Jue Liu

**Affiliations:** 1 Center for Health Policy Research and Evaluation, Renmin University of China, Beijing, 100872 China; 2 School of Public Administration and Policy, Renmin University of China, Beijing, 100872 China; 3 Department of Epidemiology and Public Health, University College London, London, WC1E 7HB UK; 4 Department of Epidemiology and Biostatistics, School of Public Health, Peking University Health Science Centre, Beijing, 100191 China; 5 Institute for Global Health and Development, Peking University, Beijing, 100871 China; 6 Key Laboratory of Reproductive Health, National Health Commission of the People’s Republic of China, Beijing, 100083 China

**Keywords:** Vaccination inequalities, Basic public health services program, Internal migrants, China

## Abstract

**Background:**

Vaccination is proved to be one of the most effective and efficient way to prevent illness and reduce health inequality. Studies about association between vaccination inequalities in the childhood and awareness of basic public health services program among internal migrants in China are lacking. In this study, we aimed to explore the association between migrants’ vaccination status between 0 and 6 years old and their awareness of the National Basic Public Health Services (BPHSs) project in China.

**Methods:**

We included 10,013 respondents aged 15 years old or above of eight provinces from 2017 Migrant Population Dynamic Monitoring Survey in China, a nationwide cross-sectional study. Univariate and multivariable logistic regressions were used to assess vaccination inequalities and the awareness of public health information.

**Results:**

Only 64.8% migrants were vaccinated in their childhood, which is far below the goal of national requirement of 100% vaccination. This also indicated the vaccination inequalities among migrants. Female, the middle-aged, married or having a relationship, the highly educated and the healthy population had higher awareness of this project than others. Both univariate and multivariate logistic regressions showed greatly significant association between vaccination status and some vaccines. Specifically, after adding convariates, the results showed that there were significant associations between the vaccination rates of eight recommended vaccines in the childhood and their awareness of BPHSs project (all p values < 0.001), including HepB vaccine (OR: 1.28; 95%CI: 1.19, 1.37), HepA vaccine (OR: 1.27; 95%CI: 1.15, 1.41), FIn vaccine (OR: 1.28; 95%CI: 1.16, 1.45), JE vaccine (OR: 1.14; 95%CI: 1.04, 1.27), TIG vaccine (OR: 1.27; 95%CI: 1.05, 1.47), DTaP vaccine (OR: 1.30; 95%CI: 1.11–1.53), MPSV vaccine (OR: 1.26; 95%CI: 1.07–1.49), HF vaccine (OR: 1.32; 95%CI: 1.11, 1.53), except for RaB vaccine (OR: 1.07; 95%CI: 0.89, 1.53).

**Conclusions:**

The vaccination inequalities exist among migrants. There is a strong relationship between the vaccination status in the childhood and the awareness rate of BPHSs project among migrants. From our findings we could know that the promotion of vaccination rates of the disadvantaged population such as the internal migrants or other minority population can help them increase the awareness of free public health services, which was proved to be beneficial for health equity and effectiveness and could promote public health in the future.

**Supplementary Information:**

The online version contains supplementary material available at 10.1186/s12889-023-16147-z.

## Introduction

Vaccination is found to be the most effective and efficient way for preventing illness [1]. In developing countries such as China, Bangladesh, India and others, it is more important to promote vaccination to reduce the spread of infectious diseases [2, 3]. Previous studies have proved that vaccination had positive impact on public health [4]. In order to promote public health, since the founding of People’s Republic of China, China has started various vaccination campaigns. Before 1978 China entered the stage of mass vaccination campaign. From 1978 to 2007, China began the stage of immunization program. Since 2007, the era of the expanded national immunization program has begun. In this immunization program, China had integrated these vaccines into the national expanded program on immunization and provided free vaccination for children [1].

Residents aged from 0 to 6 years old have been asked to be vaccinated with HepB vaccine, BCG vaccine, polio vaccine, DPT vaccine, measles vaccine, HepA vaccine, meningitis vaccine, encephalitis vaccine, measles vaccine and other national immunization program vaccines [1, 5]. As children below 6 years old may not have ability to get vaccinated initiatively, the community asked their parents bring their children to the local healthcare center to get vaccinated. In China, the primary medical and health service institutions are set to provide basic healthcare services. The community health service center and community health service station in urban areas and township hospitals and village clinics in rural areas are institutions to provide vaccination services [6, 7].

In the recent years, in order to promote people’s health status, Chinese government had introduced tons of policies, and the National Basic Public Health Services (BPHSs) project was one of these [8, 9]. It has been more than ten years since China began to implement this project, which was set to provide equitable basic healthcare services for residents in any area. As one of the key reform policies of the “new medical reform” in 2009, and based on the practical challenges at that time, this project covered maternal and child health care, elderly health care, chronic disease management, vaccination, health education, and gradually became the main source of income for primary care institutions [10]. People can enjoy these healthcare services with no cost from themselves for the national finance and local finance have paid for it. The service quality and financial support of BPHSs project had continuously improved during the past years, and this project had promoted the equalization of basic health services in China.

There were several literatures on BPHSs project in previous studies. The exiting studies had focused on the equalization, financing, and performance appraisal of BPHSs projects [11–14]. In terms of service content, previous studies focused on the management of diabetes and hypertension, the improvement of health literacy, and the establishment of health files, while less attention was paid to the awareness of this program and people’s health behavior in their early years [13, 15, 16]. In these research, BPHSs project was proved to relate to effective control of chronic diseases [11, 17], the establishment of health records [18] and so on.

As to the awareness of BPHSs project, scholars did much research based on national or local conditions [12, 17, 19]. For example, Xu et al. conducted a survey of 733 residents in Anhui Province, and the results showed that the awareness rate of the BPHSs project was 21.86% [20]. Hao et al. found that the overall awareness rate of Guangdong residents about the free service policy of national basic public health service items was 13.52% (284/2100) [21]. Guo et al. selected Beijing, Shanghai, and Shenzhen as the research sites. In each city 2 districts, 5 streets in each district, 100 internal migrants in each street were randomly selected as the research objects, and 2504 people were effectively investigated. The study found that the awareness rate of basic public health services of the internal migrants was low, at 37.5%. Among all the projects, the awareness rate of vaccination was the highest, as high as 34.4% [22]. However, compared with other aspects, present research paid less attention to the association between vaccination in their early years and the awareness of BPHSs project.

Internal migrants (hereafter as migrant) are defined as individuals who migrate between regions within one country [23, 24]. According to the seventh national census of China, the total number of internal migrants in China was 375.82 million in 2020, which increased 69.73% compared with the data of the sixth national census of China in 2010 [23]. Studies show that these migrants experience acculturation since China has significant disparities in culture, economic development, social environment and other aspects across regions [24, 25]. Therefore, paying special attention to the migrant workers is of great significance to promote health inequality. Although there were studies focused on the awareness of BPHSs project among migrants, they only did research on the developed areas (such as Beijing, Shanghai, Guangzhou, etc.) and lacked the analysis of the overall status of the migrants across the country.

Based on the intergenerational transmission theory, parents’ health knowledge has impact on their children’ health both in the childhood and after they became adults [26–29]. However, there is little evidence about the association between vaccination health knowledge, attitudes and behaviors in the childhood and the awareness and utilization of health services in the adulthood among migrants in China. In this study, we aimed at studying the vaccination status of the migrants nationwide and the awareness of the BPHSs project. The most important thing was that we would analyze the relationship between the awareness of BPHSs project and vaccination behavior in the early years of their lives through this research by using the intergeneration transmission framework. And we proposed policy recommendations for increasing the awareness and utilization of healthcare services (especially free healthcare services) through improving the health attitudes and behavior in the early life. We supposed that the government and healthcare institutions to promote health services and health education for adults especially parents with children below six years old, which was helpful for reducing disparities in health and improving public health in the future.

## Materials and methods

### Data collection and study design

In this study, we used data from 2017 Migrant Population Dynamic Monitoring Survey (MDMS), which was a nationally representative demographic and health survey of migrant population conducted by National Health and Family Planning Commission of the People’s Republic of China (NHFPC). The China Population and Development Research Center were responsible for survey design, sampling design and sampling, index system and questionnaire design, training, investigator training, on-site supervision, data reporting, data cleaning, and data summary and analysis. The National Health Commission was responsible for data management and distribution. The China Center for Disease Control and Prevention was responsible for questionnaire design, survey training and data analysis in key areas of population health. The sample of this survey were Chinese internal migrants who were or older than 15 years old when the study was conducted, and it required that the interviewee must live in a place other than the place of residence for more than one month. All data could be obtained from the website online after permission (https://www.ncmi.cn/phda/dataDetails.do?id=CSTR:A0006.11.A000T.201906.000225).

According to the needs of the management of the health and family planning services of the migrants and policy researchers, based on the principle of randomization, sample sites were drawn from the 31 provinces (autonomous regions, municipalities) and the inflow areas of the Xinjiang Production and Construction Corps. The sampling process insured that the sample provinces included developed and developing areas. Sampling surveys were carried out to obtain the results which would be representative of the whole country and all provinces. At the same time, this survey also selected eight representative cities (states, districts) to conduct a special survey on the key infectious diseases of the migrants. The selection process insured those provinces in the east, middle and west parts of China were all contained. As the results shown, Jiangsu province, Guangdong province and Shandong province in the eastern China, Henan province and Hunan province in the middle China, Chongqing, Xinjiang and Yunnan province in the western China were chosen as sample cities.

**Fig. 1:**
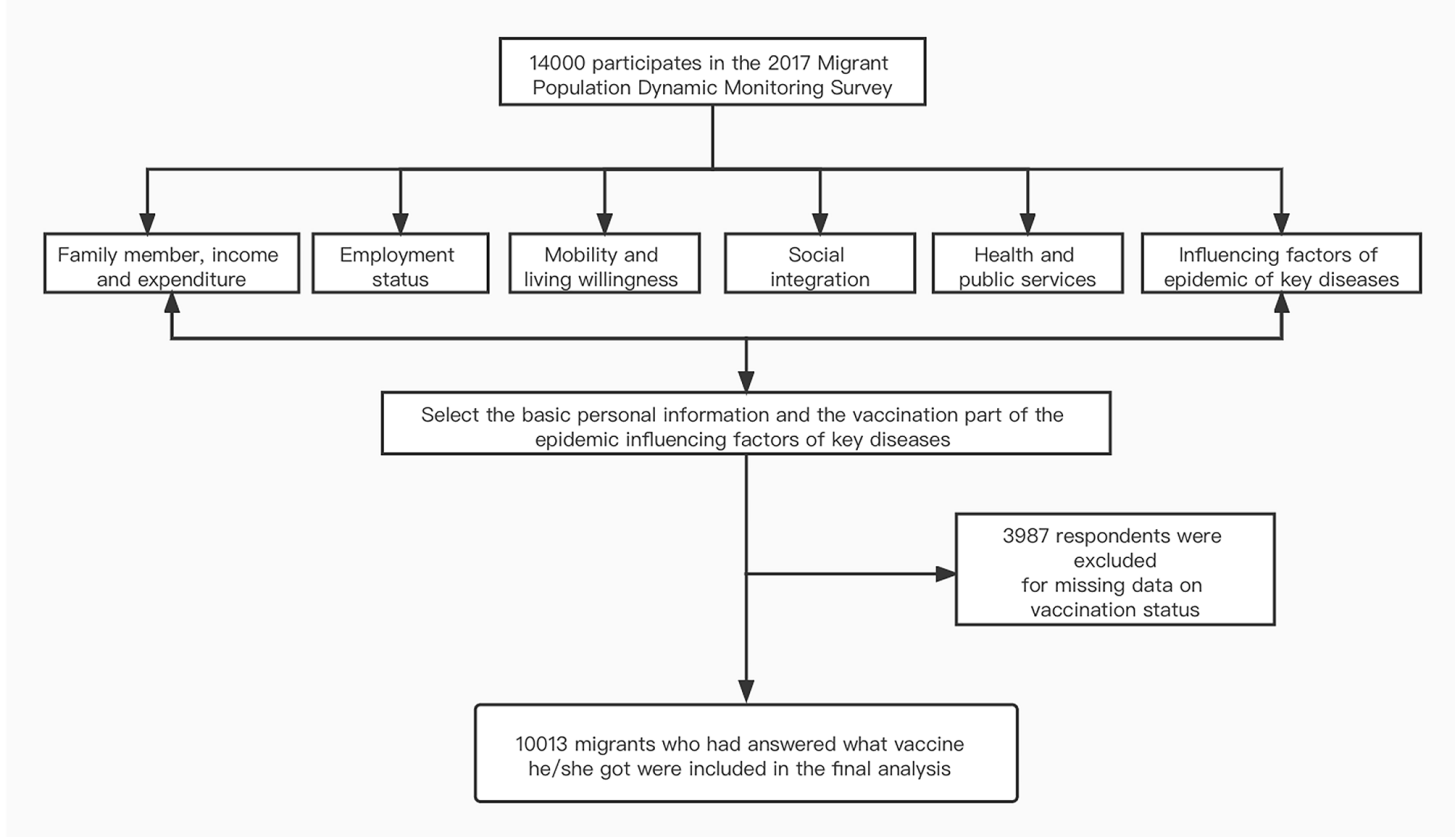
The flow chart of the respondents

In this paper, we mainly used the special survey which were about the key infectious diseases of migrants and this survey involved 14,000 samples. In order to make the results clearer, we dropped the data which answer was unknown to the vaccination questions. After removing 3987 samples who did not know their vaccination status, we got a total of 10,013 samples (Fig. 1). The basic characteristics of the missing samples were similar to the 10,013 samples (Supplemental Table 1). The investigators experienced three stages when selecting the samples: (1) Select townships (towns, streets) according to the probability proportional to size sampling method (PPS). (2) Select residents in the selected townships (towns, sub-districts). (3) Select individual survey subjects from the selected communities. The flow chart showed the selection of the respondents of this study (Fig. 1).

### Measures

After confirming the samples, on-site investigators (including investigation instructors and investigators) would begin to collect the questionnaire. Before the investigation work officially began, all investigators were required to receive training courses. All investigators need to participate in investigation training classes and receive special training. This survey was conducted via mobile phones or pads, investigators need to ask questions one by one in the order of the questionnaire and select the corresponding option on the device based on the respondent’s answer. After the survey, investigators should be organized to conduct quality check work to ensure the accuracy of the answers to the questionnaire.

According to the questionnaire, this survey included demographic information, social economic status, migration and mobility experience, medical and health services, marriage and childbirth, etc. In 2017 MDMS survey, there were factors affecting the prevalence of essential diseases, which included the vaccination information, living environment, living habits and so on.

#### Vaccination rates

The outcome of this study was whether the respondent was vaccinated in their childhood, and there were nine vaccines asked in detail (HepB, HepA, FIn, DTaP, JE, RaB, MPSV, HF, and TIG vaccine). The participants in this survey were asked each vaccine respectively to ensure all nine vaccines could have a separate outcome. The answers were yes, no, and unknown. Respondents were told to recall their vaccination status in the childhood. The vaccination records kept by some respondents provided an effective reference for answering this question. And the vaccination rate was calculated by dividing the number of people who were vaccinated by the total population and multiplying by 100%.

#### The awareness of BPHSs program

The awareness of BPHSs program was asked by the question “Have you heard of the National Basic Public Health Services Program?”. The response was yes or no, which meant “have heard” or “not have heard”. And the awareness rate of BPHSs program was calculated by dividing the number of people who were aware of the BPHSs program by the total population and multiplying by 100%.

#### Demographic characteristics

In this research, we used six characteristics as independent variables (five were demographic characteristics and one was health characteristic): gender (male/ female); age ( ≦ 30, 31–40, 41–50, > 50); marital status (single, married or having a relationship, divorced or widowed); region (rural or urban); education (middle school or below, high school, three-year technical college, university or above); health status (healthy, almost healthy, unhealthy).

### Statistical analysis

Stata version 14.0 was used as the data analysis software to conduct the statistical analysis. In terms of descriptive statistics, this article used frequency, proportions, and chi-square tests to analyze the characteristics of the sample and the awareness of BPHSs project. The frequency and proportions showed the demographic information and health level of these migrants while the p value of Chi-square tests showed the association between each characteristic and the awareness of BPHS project.

In addition, as the measurement of the awareness of BPHSs project and vaccination rates of each vaccine were both dummy variables, we used logistic regressions to analyze the relationship between demographic or health characteristics and awareness of BPHSs project as well as the vaccination rates. And we also controlled other variables, including gender, age, marital status, region, education and health status. Results from logistic regressions were presented as odds ratio (OR) and 95% confidence intervals (CI). P < 0.05, p < 0.01, p < 0.001 represented different degrees of statistical significance.

Model 1 was a univariate model which contained no adjustment (Table 1). In model 2, we adjusted for basic demographic characteristics including gender, age, marital status, region, education, and health status (Table 2). To examine the robust of the results, we conducted a sensitivity analysis by fitting different models. We adjusted for all potential confounding factors which included health status, health records, whether received propaganda from paper, whether received propaganda from promotional video, whether received propaganda from Internet, the time cost from the place of residence to the nearest medical service organization (including community health service center, village infirmary, hospital and so on) (Table 3).

**Table 1:** Association between BPHS awareness and vaccination in univariate models (Model 1)

	HepB	HepA	FIn	DTaP	JE	RaB	MPSV	HF	TIG
	OR (95%CI)	OR (95%CI)	OR (95%CI)	OR (95%CI)	OR (95%CI)	OR (95%CI)	OR (95%CI)	OR (95%CI)	OR (95%CI)
**Yes**	1.12 (1.01–1.22)	1.44 (1.25–1.73)	1.28 (1.13–1.44)	1.22 (1.05–1.43)	1.29 (1.12–1.54)	1.11 (0.94–1.32)	1.28 (1.19–1.51)	1.32 (1.03–1.70)	1.28 (1.11–1.48)
p	0.028*	<0.001***	<0.001***	0.010*	<0.001***	0.187	0.003**	0.032*	<0.001***

*p < 0.05, **p < 0.01, ***p < 0.001

**Table 2:** Association between BPHS awareness and vaccination in multivariate models (Model 2)

	HepB	HepA	FIn	DTaP	JE	RaB	MPSV	HF	TIG
	OR (95%CI)	OR (95%CI)	OR (95%CI)	OR (95%CI)	OR (95%CI)	OR (95%CI)	OR (95%CI)	OR (95%CI)	OR (95%CI)
BPHS awareness (Not heard as reference)									
	1.34** (1.12, 1.76)	1.69*** (1.23, 2.15)	1.30*** (1.05, 2.05)	1.47*** (1.21, 2.87)	1.79*** (1.19, 3.00)	1.57 (0.97, 2.42)	1.32** (1.04, 2.35)	1.02** (1.00, 1.27)	1.28*** (1.07, 1.54)
Health record (No health record as reference)									
	1.24*** (1.13, 1.36)	1.27** (1.09, 1.38)	1.03 (0.90, 1.18)	1.16 (0.97, 1.40)	0.99 (0.73, 1.18)	1.04 (0.87, 1.23)	1.06 (0.88, 1.29)	1.31* (1.00, 1.73)	1.00 (0.86,1.17)
Propaganda from paper (Not received propaganda from paper as reference)									
	1.14* (1.00, 1.30)	1.36** (1.14, 1.65)	1.01 (0.83, 1.24)	1.06 (0.80, 1.41)	1.10 (0.86, 1.42)	1.32 (0.99, 1.76)	1.00 (0.75, 1.35)	1.22 (0.76, 1.96)	1.09 (0.16, 0.33)
Propaganda from promotional video (Not received propaganda from promotional video as reference)									
	1.02 (0.92, 1.14)	1.05 (0.91, 1.21)	1.17 (0.99, 1.37)	1.18 (0.94, 1.49)	1.07 (0.87, 1.30)	1.53*** (1.22, 1.91)	1.65*** (1.28, 2.13)	1.70** (1.15, 2.50)	1.24** (1.02, 1.50)
Propaganda from Internet (propaganda from Internet as reference)									
	1.20*** (1.10, 1.31)	1.23*** (1.10, 1.39)	1.57*** (1.38, 1.78)	1.79*** (1.50, 2.13)	1.39*** (1.19, 1.63)	1.26** (1.07, 1.48)	1.60*** (1.34, 1.92)	1.73*** (1.33, 2.25)	1.31*** (1.13, 1.52)
Time cost from the place of residence to the nearest medical service organization (within 15 min)									
15–30 min	1.12 (1.00, 1.26)	1.02 (0.87, 1.19)	0.98 (0.82, 1.17)	0.80 (0.62, 1.04)	0.92 (0.73, 1.15)	0.85 (0.67, 1.07)	0.87 (0.67, 1.13)	1.06 (0.75, 1.51)	0.89 (0.72, 1.10)
30–60 min	0.99 (0.74, 1.32)	1.02 (0.68, 1.51)	0.67* (0.34, 0.98)	0.85 (0.46, 1.58)	1.01 (0.59, 1.72)	0.35** (0.14, 0.85)	0.42 (0.17, 1.04)	0.37 (0.09, 1.52)	0.69 (0.38, 1.24)
Above 60 min	0.24** (0.09, 0.64)	0.31 (0.74, 1.31)	0.47 (0.36, 1.32)	0.36 (0.05, 2.65)	0.55 (0.17, 1.33)	0.54 (0.18, 1.35)	0.79 (0.57, 1.03)	0.33 (0.15, 1.29)	0.43 (0.28, 1.17)
P value for the model	<0.001***	<0.001***	<0.001***	<0.001***	<0.001***	<0.001***	<0.001***	<0.001***	<0.001***

*p < 0.05, **p < 0.01, ***p < 0.001

Notes: We adjusted for all covariates by adding five healthcare services related variables. They were health records, whether received propaganda from paper, whether received propaganda from promotional video, whether received propaganda from Internet, the time cost from the place of residence to the nearest medical service organization (including community health service center, village infirmary, hospital, etc.). OR: Odds Ratio

**Table 3:** Sensitivity analysis on the association between BPHS awareness and vaccination in multivariate models (Model 3)

	HepB	HepA	FIn	DTaP	JE	RaB	MPSV	HF	TIG
	OR (95%CI)	OR (95%CI)	OR (95%CI)	OR (95%CI)	OR (95%CI)	OR (95%CI)	OR (95%CI)	OR (95%CI)	OR (95%CI)
BPHS awareness (Not heard as reference)									
	1.28*** (1.19, 1.37)	1.27*** (1.15, 1.41)	1.28*** (1.16, 1.45)	1.32** (1.11, 1.53)	1.14*** (1.04, 1.27)	1.07 (0.89, 1.53)	1.26** (1.09, 1.49)	1.21*** (1.04, 1.52)	1.27*** (1.05, 1.47)
Gender (Female as reference)									
	1.03 (0.96, 1.11)	1.08 (0.98, 1.19)	1.01 (0.91, 1.13)	0.96 (0.83, 1.11)	1.06 (0.93, 1.22)	1.50*** (1.31, 1.73)	1.07 (0.92, 1.25)	0.19 (0.02,1.12)	0.28 (0.18, 1.29)
Age ( < = 30 as reference)									
31–40	0.96 (0.88, 1.06)	0.91 (0.81, 1.03)	0.91 (0.80, 1.04)	0.91 (0.76, 1.10)	0.84* (0.71, 0.99)	0.87 (0.73, 1.04)	0.95 (0.78, 1.15)	0.82 (0.19, 1.98)	0.88 (0.72, 1.26)
41–50	0.69*** (0.62, 0.77)	0.73*** (0.62, 0.85)	0.69*** (0.58, 0.82)	0.63*** (0.45, 0.81)	0.61*** (0.49, 0.76)	0.68** (0.54, 0.85)	0.60*** (0.47, 0.78)	0.29 (0.12, 1.12)	0.92 (0.53, 1.21)
> 50	0.48*** (0.41, 0.57)	0.62*** (0.49, 0.78)	0.67** (0.52, 0.86)	0.66* (0.46, 0.95)	0.57** (0.41, 0.79)	0.53*** (0.38, 0.76)	0.67* (0.47, 0.96)	0.47** (0.36, 0.79)	0.67** (0.24, 0.86)
Marital status (Single as reference)									
Married/Having a relationship	0.77*** (0.70, 0.86)	0.78*** (0.68, 0.89)	0.72*** (0.63, 0.84)	0.76** (0.62, 0.93)	0.86 (0.72, 1.04)	0.69*** (0.57, 0.83)	0.82 (0.66, 1.01)	0.72 (0.57, 1.13)	0.74*** (0.72, 0.91)
Divorced/widowed	0.90 (0.69, 1.17)	0.77 (0.53, 1.11)	0.62** (0.40, 0.96)	0.85 (0.49, 1.46)	0.73 (0.42, 1.28)	0.85 (0.51,1.42)	1.10 (0.65, 1.87)	0.22 (0.10, 1.25)	0.98 (0.73, 1.21)
Region (Rural as reference)									
Urban	0.88* (0.80, 0.97)	0.95 (0.83, 1.09)	0.92 (0.79, 1.06)	0.95 (0.78, 1.67)	0.94 (0.78, 1.13)	0.94 (0.78, 1.14)	1.03 (0.83, 1.26)	0.97 (0.83, 1.27)	0.73 (0.54, 1.18)
Education attainment (Middle school or below)									
High school	1.42*** (1.30, 1.54)	1.23** (1.09, 1.38)	1.32*** (1.17, 1.50)	1.11 (0.93, 1.32)	1.09 (0.93, 1.28)	1.22* (1.03, 1.44)	1.10 (0.92, 1.32)	1.12** (1.06, 1.27)	1.23* (1.07, 1.56)
Three-year technical college	1.73*** (1.54, 1.94)	1.19* (1.02, 1.39)	1.23* (1.04, 1.45)	1.10 (0.88, 1.39)	1.02 (0.82, 1.26)	1.28* (1.03, 1.58)	0.92 (0.72, 1.19)	1.08* (1.02, 1.17)	1.22 (0.87, 1.59)
University or above	1.78*** (1.53, 2.07)	1.05 (0.85, 1.28)	1.24* (1.00, 1.54)	0.75 (0.54, 1.04)	1.03 (0.78, 1.35)	1.13 (0.86, 1.50)	1.04 (0.76, 1.42)	1.12 (1.05, 1.37)	1.07 (0.73, 1.28)
Health status (Healthy as reference)									
Unhealthy	0.84** (0.75, 0.93)	0.83* (0.72, 0.97)	0.74** (0.63, 0.88)	0.67** (0.52, 0.86)	0.72** (0.57, 0.90)	0.98 (0.79, 1.21)	0.79 (0.62, 1.02)	0.89 (0.72, 1.03)	0.75** (0.54, 1.23)
Whether pay attention to the window is opened for ventilation (No for reference)									
Sometimes	1.13 (0.56, 2.30)	0.48 (0.22, 1.06)	0.89 (0.31, 2.52)	0.78 (0.19, 3.29)	2.06 (0.28, 15.12)	0.82 (0.19, 3.44)	0.77 (0.18, 3.25)	0.85 (0.19, 2.74)	0.89 (0.46, 1.22)
Everyday	1.17 (0.58, 2.37)	0.56 (0.26, 1.23)	0.93 (0.33, 2.64)	0.91 (0.22, 3.81)	2.34 (0.32, 17.16)	1.16 (0.28, 4.86)	0.86 (0.21, 3.59)	1.12 (0.89, 1.58)	1.18 (1.03, 1.72)
Whether use the air purifier in the daily life (No for reference)									
	1.40*** (1.23, 1.60)	1.39*** (1.17, 1.64)	1.58*** (1.33, 1.88)	2.06*** (1.66, 2.57)	1.97*** (1.61, 2.42)	1.85*** (1.49, 2.28)	1.78*** (1.41, 2.26)	1.99*** (1.47, 2.73)	1.79 (0.69, 1.83)
P value for the model	<0.001***	<0.001***	<0.001***	<0.001***	<0.001***	<0.001***	<0.001***	<0.001***	<0.001***

*p < 0.05, **p < 0.01, ***p < 0.001

Notes: We adjusted for six demographic and health characteristics, including gender, age, marital status, region, education, health status, whether pay attention to the window is opened for ventilation and whether use the air purifier in the daily life.

## Results

### Sample characteristics

The descriptive results showed people with different demographic and characteristic characteristics had different health knowledge, which was reflected by the awareness of the awareness of BPHSs program. In this survey, people aged from 15 years old to 73 years old were all contained. The mean of age is 36.73 years old, and the median of age is 33 years old. Among the total sample, 7658 (76.48%) samples indicated that they had heard of the BPHSs program, and 2355 (23.52%) indicated that they had not heard of it, which showed that most migrants in China knew this health promoting program.

As to gender, the percentages of the two genders were similar while the awareness rate was higher in women (78.47%) rather than men (74.51%), and the p value of chi-square test also reflected that female were more likely to know the BPHS project than male (P value < 0.001). From the perspective of age, it showed that migrants between 31 and 40 years old had highest awareness rate of the BPHSs project. People who were married or having a relationship took up the largest part in this survey, accounting for 7538 (75.28%). In this group, people who knew about this project are about four times as many people who don’t know about this project.

The number of migrants from rural areas was much larger than those from urban areas, which showed that the migrants were mostly moving from village to city. Among the migrants with household registration in rural areas, 76.66% of them were aware of this project, which was nearly three times the number of people who had not known this project while there were only twice in urban household registration. In terms of education attainment, the awareness of BPHSs projects was basically proportional to the level of education, which approximately showed that the higher education level people get, the higher awareness rate of BPHSs program. In terms of health level, among these people, the healthy population has the highest proportion of knowledge about the project (Table 4).

**Table 4:** Characteristics of the respondents according to awareness of BPHSs project at the baseline

Characteristics	N (%)	Awareness of BPHSs Project				χ^2^	P value
		Yes (%)	No (%)	OR	95%CI		
**Total**	10,013 (100)	7658 (76.48)	2355 (23.52)				
**Gender**						21.8072	<0.001
Male	5021 (50.14)	3741 (74.51)	1280 (24.90)	1.00	-		
Female	4992 (49.86)	3917 (78.47)	1075 (21.53)	0.93	0.89, 0.97		
**Age**						49.7791	<0.001
<=30	4294 (42.88)	3159 (73.57)	1135 (26.43)	1.00	-		
31–40	3155 (31.51)	2516 (79.75)	639 (20.25)	1.37	1.03, 1.81		
41–50	1916 (19.14)	1511 (78.86)	405 (21.14)	1.89	1.36, 2.63		
> 50	648 (6.47)	472 (72.84)	176 (27.16)	1.25	1.04, 1.97		
**Marital status**						71.1088	<0.001
Single	2127 (21.24)	1484 (69.77)	643 (30.23)	1.00	-		
Married/Having a relationship	7538 (75.28)	5916 (78.48)	1622 (21.52)	1.09	1.21, 1.47		
Divorced/widowed	348 (3.48)	258 (74.14)	90 (25.86)	1.13	1.02, 1.41		
**Region**						13.8164	<0.001
Rural	9891 (98.78)	7582 (76.66)	2309 (23.34)	1.00	-		
Urban	122 (1.22)	76 (62.30)	46 (37.70)	1.28	1.19, 1.37		
**Education**						93.4021	<0.001
Middle school or below	2144 (21.41)	1492 (69.59)	652 (30.41)	1.00	-		
High school	2712 (27.08)	2044 (75.37)	668 (24.63)	1.21	1.03, 1.92		
Three-year technical college	2948 (29.44)	2370 (80.39)	578 (19.61)	1.98	1.45, 2.71		
University or above	2209 (22.06)	1752 (79.31)	457 (20.69)	1.69	1.20, 2.39		
**Health status**						22.2457	<0.001
Healthy	8563 (85.52)	6606 (77.15)	1957 (22.85)	1.00	-		
Almost healthy	1279 (12.77)	942 (73.65)	337 (26.35)	1.10	1.05, 1.42		
Unhealthy	171 (1.71)	110 (21.65)	398 (78.35)	1.38	1.11, 1.73		

*p < 0.05, **p < 0.01, ***p < 0.001

### Association between characteristics and the awareness of BPHSs project

In this research, group of males, below or equal to 30 years old, single, rural, middle school or below, healthy population were set as the reference groups. From the chi-square test results (Table 4), we found that all the characteristics we analyzed were significantly associated with migrants’ awareness of BPHSs project (all p values < 0.001). The connection between gender and the awareness was significant (OR: 0.93, 95%CI: 0.89, 0.97). Results also showed that the percentage of people who were aware of the BPHSs project was higher among those who were married or having a relationship (OR: 1.09, 95%CI: 1.21, 1.47), from urban areas (OR: 2.00, 95%CI: 1.19, 1.37), almost healthy (OR: 1.10, 95%CI: 1.05, 1.42) (Table 4).

### Awareness rates of basic public health services among migrants vaccinated

**Fig. 2:**
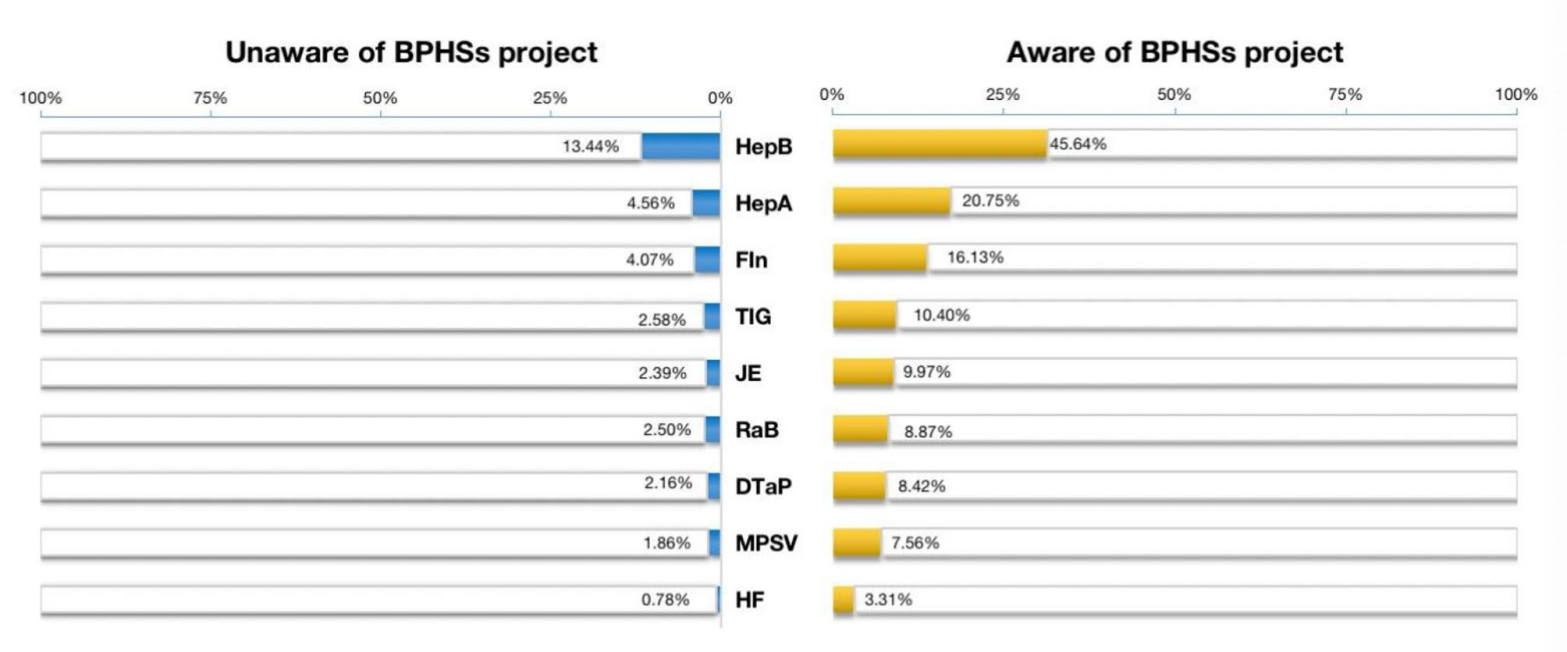
Vaccination rates among migrants who were aware or unaware of the BPHSs project

We tested the frequency and proportions of the awareness rates of BPHSs project among migrants who had been already vaccinated with one of the nine recommended vaccines at least. And at the same time, we analyzed the vaccination rates among the migrants who were aware or unaware of the BPHSs project (Fig. 2). The number of individuals who had known the BPHSs project among the vaccinated people were almost 3 to 4 times than those had not. Among the HepA, HF, JE, MPSV, TIG vaccine there were more than 80% people who were conscious of this project, accounting for 81.97%, 80.93%, 80.68%, 80.28%, 80.14%, respectively. Others were nearly to 80%, and people taking FIn vaccine and had learned the project took up for 79.83%, while the DTaP vaccine was 79.60%, the RaB vaccine was 78.03% and the HepB vaccine was 77.25% (Fig. 2).

### The association between vaccination rates and awareness rates of BPHSs project

In order to figure out the association between vaccination rates of nine recommended vaccines and the awareness rates of the BPHSs project, we conducted logistic regressions. The group who had not known the BPHSs project was set as the reference group to compare with people who had heard it. The results showed that people with higher vaccination rates were more likely to have heard of the BPHSs project. There were significant association between the vaccination and awareness of BPHSs project in HepA vaccine (OR: 1.69, 95%CI: 1.21–2.15), FIn vaccine (OR: 1.30, 95%CI: 1.05–2.05), JE vaccine (OR: 1.79, 95%CI: 1.19-3.00), TIG vaccine (OR:1.28, 95%CI: 1.07–1.54), and DTaP (OR: 1.47, 95%CI: 1.21–2.87) (all p values < 0.001). The significance in HepB (OR: 1.34, 95%CI: 1.12–1.76), MPSV (OR: 1.32, 95%CI: 1.04–2.35), and HF (OR: 1.02, 95%CI: 1.00-1.27) was slighter (p < 0.05) in the multivariate models (Table 2). Moreover, according to the sensitivity analysis, the results were robust in different models (Table 3).

## Discussion

To our knowledge, this is the first nationwide cross-sectional study which examined the association of between vaccination status in the childhood and awareness of basic public health services among migrants aged 15 years old or above in China. We found that there was a significant relationship between the BPHSs project and vaccination behavior in the early life. In addition, the demographic and health characteristics were highly associated with the awareness of BPHSs project.

Chinese government had introduced the “Suggestions on Further Strengthening the Services and Management of the Migrants” in 2007, which said that the number of the migrants continued to grow, and the services and management of the migrants should be strengthened. The government need to pay great attention to medical, health and other services for the migrants, and provide free services equivalent to those of the local registered population in terms of disease prevention, infectious disease prevention, vaccination, and maternal and child health care. The document emphasized that the migrants could enjoy the same preferential policies as other citizens.

From the perspective of the association between the basic information and health status of the BPHSs project, the migrants’ awareness rates of the BPHSs project were significantly related to gender, age, marital status, region, education level and health status. Specifically, in the division of labor in traditional Chinese families, women were more responsible for children’s vaccination, so women would have a better understanding of vaccination and basic public health services in the process.

In regard to age, there was a strong and significant relationship between age and the awareness of BPHSs project. One possible explanation was these 31–40 and 41–50 groups were in the middle age group and had more channels for obtaining information than the elderly group. At the same time, they had a higher level of awareness of social affairs than the young group. From the perspective of marital status, the vaccination rates of married or having relationship was higher than that of other groups and this might also be related to the needs of children.

The relationship between regions and the awareness of BPHSs project might because that migrants in urban areas had less contact with the community health service centers responsible for providing basic public health services. In regard to education level, as a whole, the higher the level of education, the higher the awareness rate of BPHSs project. This was related to the higher level of education that paid more attention to their own health status and related health services. As to health level, the higher the health level, the higher the awareness rate of the BPHSs project. This article conjectured that this reflected the mutual promotion relationship between the two aspects.

The awareness rate of BPHSs project of migrants in the present study was 76.48%, which was much higher than the results of previous studies [22]. We believe the health publicity and education have played a great role. The correlation between the awareness rate of BPHSs project and vaccination in the childhood might because parents have carried out better health intervention on children in their childhood. This kind of good health behavior is passed on from generation to generation through health intervention, thus affecting their health behavior and cognitive ability in adulthood. By receiving vaccination at the vaccination sites, the migrants had more opportunities to receive the publicity of health services, which may also influence their health knowledge after they grown up. The close correlation between this project and vaccination may imply that could improving vaccination rates in the childhood may strengthen the awareness of BPHSs project, thereby promoting the development of national health program and improving the health literacy and status of residents [12, 30]. Research had shown that there was a correlation between parents and children regarding to health behavior. Based on these research, health knowledge transmitted from parents to children and the early intervention is significant for children to cultivate good health knowledge, which could help to develop their healthy lifestyles in their later lives [26–29]. Promoting health knowledge attainment in the childhood thorough parents was an effective way to help children cultivate their good health habits and strengthen their health utilization in their adulthood.

There were several practical, theoretical, and managerial implications for our research. First of all, we firstly tried to link the childhood vaccination behavior with adult vaccination behavior of migrants, which could provide support for promoting health behavior of adults. Second, this research used intergenerational transmission theory to explain the impact of parental health awareness and health behavior on their children’ health behavior. The application of this theory has made a certain contribution to the improvement of health level from the sociological field. Furthermore, from our research we argue that government and related healthcare institutions should pay more attention to cultivate children’s health awareness through parents’ health behavior, so as to promote the improvement of public health.

Our research certainly had some limitations. Firstly, this study could only explain the relationship between the awareness of BPHSs project and vaccination status in the childhood but cannot explain the causal relationship between them. The awareness of BPHS program may also cause the migrants to get vaccinated in the reverse direction. This issue needed further research. Secondly, the data in this article were second-hand data. Many factors (such as health literacy, parental education, etc.) were not available, which might affect the results of the research. Thirdly, all data in this research were self-reported data, in which both dependent and independent variables were measured by using the self-rated health indicators, which were not objective indicators. This self-assessment of health indicators may lead to the problem of homologous bias.

In order to strengthen the research on the awareness rates of the BPHSs project and the childhood vaccination of the migrants, we planned to study the causal relationship between the project and vaccination in the future, using the latest data in the research process. At the same time, we would like to study the vaccination status of migrants’ children and their awareness of BPHSs besides the adults. At the same time, using data from public healthcare institutions like community healthcare center, township hospitals and village clinics and self-reported data to analyze the association between vaccination rates and BPHSs project.

In the future, with regards to improve people’s health awareness and health behavior, we need to pay attention to the socioeconomic determinants and try to influence people’s health behavior in the adulthood from their childhood [23]. We need to take health as a long-term process and promote public health in the long run.

## Conclusion

In this study, we found that there was a strong relationship between vaccination in the childhood and the awareness rate of BPHSs project among migrants, and the awareness rate of BPHSs had a strong correlation with the demographic and health characteristics of the migrants. Our findings suggest that the promotion of vaccination rates of the disadvantaged population like internal migrants or other minority population in the childhood can help them increase the awareness of free public health services, which was proved to be beneficial for health equity and effectiveness and improve the overall level of public health. Future research should explore the mechanism of promoting vaccination coverage rates on the awareness of healthcare services.

## Electronic supplementary material

Below is the link to the electronic supplementary material.

Supplementary Material 1

## Data Availability

Data are available from the corresponding author by request.

## Abbreviations

BPHSs: National Basic Public Health Services
HepA: Hepatitis A vaccine
FIn: Influenza vaccine
JE: Japanese encephalitis vaccine
TIG: Tetanic immunity globulin vaccine
HepB: Hepatitis B vaccine
DTaP: Diphtheria and tetanus toxoid with acellular pertussis vaccine
MPSV: Meningococcal polysaccharide vaccine
HF: Hemorrhagic fever vaccine
RaB: Rabies vaccine
OR: Odds Ratio
95%CI: 95% Confidence Interval
