# Supplementary material for: Vaccination in the childhood and awareness of basic public health services program among internal migrants: a nationwide cross-sectional study
Source: BMC Public Health. 2023 Jun 28;23:1257. doi: 10.1186/s12889-023-16147-z (PMC10308709; doi:10.1186/s12889-023-16147-z)
Supplement: Supplementary file 1 — Supplementary Material 1 [file 12889_2023_16147_MOESM1_ESM.docx]

**Supplemental Table 1 Characteristics of the missing samples**

| **Characteristics** | **N(%)** | **Awareness of BPHSs Project** | | | | **χ^2^** | **P value** |
| --- | --- | --- | --- | --- | --- | --- | --- |
|  |  | **Yes(%)** | **No(%)** | **OR** | **95%CI** |  |  |
| **Total** | 3987 (100) |  |  |  |  |  |  |
| **Gender** | | | | | | 74.2190 | 0.000 |
| Male | 2091 (52.44) | 1379 (65.95) | 712 (34.05) | 1.00 | - |  |  |
| Female | 1986 (47.56) | 1335 (67.22) | 651 (32.78) | 0.89 | 0.76, 0.93 |  |  |
| **Age** | | | | | | 52.0194 | 0.000 |
| <=30 | 2041 (51.19) | 1359 (66.59) | 682 (33.41) | 1.00 | - |  |  |
| 31-40 | 987 (24.76) | 579 (58.66) | 408 (41.34) | 1.28 | 1.02, 1.73 |  |  |
| 41-50 | 664 (16.65) | 425 (64.01) | 239 (35.99) | 1.44 | 1.17, 1.79 |  |  |
| >50 | 295 (7.40) | 133 (45.08) | 162 (54.92) | 1.35 | 1.06, 1.65 |  |  |
| **Marital status** | | | | | | 69.1083 | 0.000 |
| Single | 1383 (34.69) | 894 (64.64) | 489 (35.36) | 1.00 | - |  |  |
| Married/Having a relationship | 2495 (62.58) | 1879 (75.31) | 616 (24.69) | 1.36 | 1.19, 1.48 |  |  |
| Divorced/widowed | 109 (2.73) | 76 (69.72) | 33 (30.28) | 1.02 | 1.00, 1.07 |  |  |
| **Region** | | | | | | 24.8724 | 0.000 |
| Rural | 3199 (80.24) | 2780 (86.90) | 419 (13.10) | 1.00 | - |  |  |
| Urban | 788 (19.76) | 675 (85.66) | 113 (14.34) | 1.76 | 1.32, 1.89 |  |  |
| **Education** | | | | | | 78.9617 | 0.000 |
| Middle school or below | 899 (22.55） | 625 (69.52) | 274 (30.48) | 1.00 | - |  |  |
| High school | 974 (24.43) | 694 (71.25) | 280 (28.75) | 1.35 | 1.17-1.86 |  |  |
| Three-year technical college | 1032 (25.88) | 852 (82.56) | 180 (17.44) | 1.74 | 1.35-2.13 |  |  |
| University or above | 1082 (27.14) | 763 (70.52) | 319 (29.48) | 1.63 | 1.21-1.97 |  |  |
| **Health status** | | | | | | 17.5632 | 0.000 |
| Healthy | 3195 (80.14) | 2741 (85.79) | 454 (14.21) | 1.00 | - |  |  |
| Almost healthy | 632 (15.85) | 479 (75.59) | 153 (24.21) | 1.17 | 1.03-1.37 |  |  |
| Unhealthy | 160 (4.01) | 108 (67.50) | 52 (32.50) | 1.05 | 1.01-1.54 |  |  |

*p<0.05, **p<0.01, ***p<0.001
